# Predictors of revision endoscopic sinus surgery in Finnish patients with chronic rhinosinusitis with nasal polyps

**DOI:** 10.1002/clt2.70032

**Published:** 2025-01-29

**Authors:** Sanna Toppila‐Salmi, Annina Lyly, Johanna Simin, Juhani Aakko, Helga Haugom Olsen, Lauri Lehtimäki

**Affiliations:** ^1^ Department of Otorhinolaryngology University of Eastern Finland Kuopio Finland; ^2^ Department of Otorhinolaryngology Wellbeing Services County of Pohjois‐Savo Kuopio Finland; ^3^ Department of Allergology Inflammation Center Helsinki University Hospital and University of Helsinki Helsinki Finland; ^4^ AstraZeneca Nordics Medical Affairs Espoo Finland; ^5^ AstraZeneca Nordics Medical and Regulatory Oslo Norway; ^6^ Medaffcon Oy Espoo Finland; ^7^ Faculty of Medicine and Health Technology Tampere University Tampere Finland; ^8^ Allergy Centre Tampere University Hospital Tampere Finland

**Keywords:** chronic rhinosinusitis, CRSwNP, endoscopic surgery, nasal polyps, revision surgery

## Abstract

**Background:**

Although patients with chronic rhinosinusitis with nasal polyps (CRSwNP) may benefit from endoscopic sinus surgery (ESS), some patients will experience polyp recurrence, adding to the overall disease burden of CRSwNP. We aimed to investigate predictors of revision ESS in patients with CRSwNP.

**Methods:**

A nationwide population‐based study including all adults diagnosed with CRSwNP who had surgical procedure codes for ESS (*N* = 3506), followed up between January 2012 and December 2019. Logistic regression models provided adjusted odds ratios (OR) with 95% confidence intervals (CIs) for the odds of revision surgery within one and 3 years post‐index surgery.

**Results:**

559 (15.9%) of the patients had at least one revision surgery during the follow‐up. Median time to revision of ESS was 425 days (interquartile range: 213–898). Baseline asthma (OR = 1.58, 95% CI 1.17–2.12) and antibiotic use (OR = 1.61, 95% CI 1.27–2.04) were associated with higher odds of revision ESS, particularly within 3 years post‐index surgery, whereas Increasing age was inversely associated with the odds of ESS revision (OR = 0.82, 95% CI 0.76–0.88). The highest odds of revision ESS were observed within 3 years post‐index surgery in patients who had undergone extensive surgery at index (OR = 14.13, 95% CI 3.41–95.64) compared with those who had undergone limited surgery. OCS use was frequent among CRSwNP patients, with a higher cumulative dose in patients undergoing multiple ESS revisions (63%, *n* = 97, median daily dose 3.29 mg, IQR: 1.64–3.70) compared with patients without revisions (49%, *n* = 1361 and 1.64 mg, IQR: 1.64–3.29, respectively. *p*‐value <0.001).

**Conclusions:**

A small proportion of CRSwNP patients require revision ESS with associated high cumulative OCS doses, highlighting the need for additional therapies to achieve disease control and reduce the corticosteroid burden. A few simple baseline characteristics can predict the need for recurrent surgery among the patients with CRSwNP.

## 1 INTRODUCTION

Chronic rhinosinusitis (CRS) is an inflammatory disease of the paranasal sinuses with distinct endotypes.^1^, ^2^ Approximately 25%–30% of all CRS patients develop nasal polyps (NP),^3^, ^4^ a benign inflammatory mass in the mucosa of the nose and paranasal sinuses.^5^, ^6^ The incidence of CRS with nasal polyps (CRSwNP) in Finland and other Nordic countries is increasing,^5^, ^7^, ^8^ and it is associated with morbidity and decreased quality of life,^3^, ^4^, ^5^, ^6^ highlighting the need for treatment options and even prevention of the disease.

Although the underlying pathophysiological mechanisms and diversity among CRSwNP are not well established, several factors are likely to play a role. Various inflammatory mechanisms in the nasal mucosa and airways, as well as genetic and environmental factors, have been linked with the development of CRSwNP.^3^, ^5^, ^9^, ^10^ Asthma is strongly associated with an increased risk of CRSwNP, and it has been estimated that nearly half (45%) of CRSwNP patients have or will develop asthma.^5^, ^9^, ^11^

In general, CRSwNP is treated with nasal corticosteroids and in case of exacerbation, oral corticosteroid (OCS) courses.^3^, ^12^ If the response to treatment with local corticosteroids is insufficient or there is a need for repeated use of OCS, endoscopic sinus surgery (ESS) may be required.^13^, ^14^, ^15^ Whilst most patients benefit from surgical treatment and achieve disease control,^16^, ^17^ some patients experience recurrent polyps leading to repeated procedures.^18^, ^19^ However, nationwide data is sparse on the predictors of revision ESS. To address this knowledge gap, we conducted a nationwide population‐based study to assess the various predictors of revision ESS and the timing of revision surgery, utilizing Finnish real‐world data.

## 2 METHODS

### 2.1 Study design

This nationwide and population‐based observational study included all Finnish adult patients with a recorded diagnosis of CRSwNP and a surgical procedure code for ESS between January 1, 2012 and December 31, 2018 (*N* = 3506), as identified from the Care Register for Healthcare (specialty care and primary care). Patients were followed up from the index date (i.e., the date of the first recorded ESS during the study period) up until December 31, 2019. However, to ensure a minimum follow‐up period of 12 months, the last possible index date was set to 31st of December 2018. This nationwide cohort utilizing real‐world data has previously been used to investigate the burden of CRSwNP and its relation to asthma in Finland.^5^

The unique Finnish personal identification number ensured an unambiguous linkage of data from the Social Insurance Institution of Finland (drug prescriptions, reimbursement numbers and drug purchases) and data from Statistics Finland (causes and dates of death). The Finnish Social and Health Data Permit Authority Findata granted the permit for the study (THL/4801/14.02.00/2020[2020/576]) by the provision of the Act on the Secondary Use of Health and Social Data (finlex 552/2019) without a need for informed consent due to the registry‐based nature of the study.

### 2.2 Exposure ascertainment

The Care Register for Healthcare (specialty care and primary care) was used to ascertain the diagnostic and procedure codes. The CRSwNP diagnoses were identified according to the International Classification of Diseases (ICD) 10^th^ revision and International Classification of Primary Care (ICPC‐2) codes, whereas the surgical procedures were identified based on the Nordic Medico‐Statistical Committee (NOMESCO) Classification of Surgical Procedures (NCSP) coding.^20^ The applied codes are presented in Table S1.

### 2.3 Exclusion criteria

To include only incident patients with nasal polyposis, patients with a diagnosis of nasal polyps during a 5‐year period prior to the study period were excluded. Also, patients with ICD‐10 codes at specialty and/or primary care (as recorded in the Care Register for Healthcare) for cystic fibrosis (E84*) or ciliary dyskinesia (Q33* and Q34*) were excluded.

### 2.4 Baseline exposure definitions

The Finnish Social Insurance Institution was used to extract data on medication purchases utilizing the Anatomical and Therapeutic Chemical (ATC) classification codes (based on the WHO Center for Drug Statistics Methodology).^21^ The exposure to intra‐nasal steroids (INCS), oral corticosteroids (OCS), antibiotics for sinusitis, inhaled asthma treatments and biological treatments were assessed up to 1 year prior to the index. For OCS and biologics (*N* < 5), their use was also assessed during the follow‐up. The ATC codes considered in this study are presented in detail in Table S2. The “defined‐daily‐dose” (DDD) per package was applied to calculate the days covered by the ICS and OCS, taking into account the potency and prescribed quantities of the drug.^22^ Regular use of ICS was defined as one‐year baseline purchases covering more than 270 days.

Based on the presence of asthma at baseline, the patients were divided into subgroups of no asthma/asthma, mild to moderate, and severe asthma. Patients were considered to have asthma if they had at least two recorded visits with an asthma diagnosis (ICD‐10 code J45) or at least two asthma controller medication purchases (ICS) in single inhalers (ATC‐codes R03BA01, R03BA02, R03BA05, R03BA07, R03BA08, R03BA09) or in combination with long‐acting beta‐2‐agonists (R03AK06, R03AK07, R03AK08, R03AK09, R03AK10, R03AK11). Severe asthma was defined applying the ERS/ATS guidelines,^23^ as daily use of fluticasone propionate 800 μg or equivalent (80% adherence to 1000 μg of fluticasone propionate or equivalent) with ≥1 other controller drug (leukotriene receptor antagonist, long‐acting beta‐agonist, long‐acting muscarinic antagonist, or biological asthma medication) (Table S2). If the criteria for severe asthma were not fulfilled, asthma was defined as mild to moderate.

Charlson comorbidity index (CCI) scoring was based on ICD‐10 codes reported up to 36 months before the index date,^24^, ^25^ and categorized as 0, 1, 2, 3, 4, 5, and more than 5. Extensive surgery at index was defined as having a recorded procedure code of DNB20 (endoscopic ethmoidectomy) with ZXC95 (navigator endoscopic technique) on the same day or codes DNB20, DPA30 (sphenotomy) and DPA25 (trephine of frontal sinus through the nose) on the same day (Table S1). Surgery not fulfilling the criteria of extended surgery was defined as limited surgery (based on the NOMESCO codes presented in Table S1).

### 2.5 Outcome ascertainment

The primary outcomes were to describe the clinical characteristics of patients requiring revision surgery and the odds of revision ESS within 1 year and 3 years from the index. The secondary outcome was to explore the impact of age, sex, drug use, type of surgery at index, CCI score and asthma on the probability of a revision ESS within 3 years from the index. A revision ESS was defined as having a visit with a recorded procedure code for ESS >90 days after the previously recorded end of an ESS episode. Subsequent visits (with a recorded ESS procedure code) that occurred within 90 days from the previous visit were merged into one episode. The applied classifications of definitions for the outcomes are presented in more detail in Table S3.

### 2.6 Statistical methods

Demographics and clinical characteristics were described at baseline, continuous variables were described by mean with standard deviation (SD) and median with interquartile range (IQR), and categorical variables by frequencies and proportions. Potential differences between populations were tested using a Wilcoxon rank‐sum test for continuous variables, and the Chi‐square test for categorical variables. *P*‐values <0.05 were considered indicative of statistical significance.

Patients were further sub‐grouped and categorized as having no revision surgery (i.e., ESS once), one ESS revision, and at least two ESS revisions after the index surgery. Predictors of repeated ESS procedures were first explored using Cox regression models, but the proportional hazard assumption was violated (data not shown here). Therefore, to evaluate the predictors of short‐term and long‐term revision ESS, two logistic regression models were used to assess the odds of revision ESS within one (1 year model) and 3 years (3‐year model) from the index, providing odds ratios (ORs) with 95% confidence intervals (CIs), and *p*‐values. For the analysis, the observations were considered independent, there was no multicollinearity among the selected independent variables and the only continuous variable, age, had a linear relationship with the log‐odds of the dependent variable. To assess multicollinearity among the independent variables, Variance Inflation Factors (VIF) were computed for each variable as well as correlation coefficients were calculated for all variable pairs. The relationship between continuous and binary variables was evaluated using the Point‐Biserial Correlation, while the relationship between binary variables was examined using the Phi Coefficient. Patients who had follow‐up times shorter than 1 year and 3 years were omitted from the 1 year and 3‐year models, respectively. Revision surgery performed during one and 3 years was included as the dependent variable in the 1 year and 3‐year models, respectively. The included independent variables in both models were age at first surgery (10 years span), sex, type of surgery at index (categorized as limited and extensive surgery at index), CCI score (categorized as 0 and 1+), asthma (binary), and use of antibiotics (binary), OCS (binary) and ICS (non‐regular and regular). In these models, the relative variable importance was based on mean Shapley values (absolute log odds scale). These values quantify the influence of each variable on the predicted log‐odds of revision surgery, in which higher (Shapley) values indicate a greater variable importance in the model.^26^, ^27^ The data was analyzed as recorded in the registers and was considered complete, so no imputation for missing values was necessary.

To further assess whether antibiotics and asthma are associated with revision ESS, the 3‐year logistic regression model was utilized to predict the probability of a revision ESS within 3 years from the index for an average patient with or without a history of asthma and with or without a history of antibiotic use as well as their combinations. To represent an average patient in the cohort, median values were applied for continuous variables and mode for categorical variables as input in the model, except for the history of asthma and antibiotic use. Confidence intervals were calculated for the predictions by bootstrapping 10,000 samples. All analyses were performed using R (4.0.3).

## 3 RESULTS

The results of this study are reported in line with the Strengthening the Reporting of Observational Studies in Epidemiology (STROBE) guidelines.^28^ A total of 3506 CRSwNP patients had at least one recorded ESS (index date), of whom 79.7% (*n* = 2794) had no revisions, 15.9% (*n* = 559) had one revision ESS, and 4.4% (*n* = 153) had undergone at least two revisions (Table 1). The overall median follow‐up time was 4.3 years (IQR: 2.70–5.96), and the median time to ESS revision was 425 days (IQR: 213–898). Patients with no revision surgery had the shortest median follow up time (4.14 years) (IQR: 2.62, 5.79) compared to patients with one‐ or at least 2 revision surgeries (median follow‐up time of (4.67 years (IQR: 3.02, 6.28) and 6.07 years (IQR: 4.54, 6.99), respectively) (Table 1). Overall, patients with CRSwNP who had undergone ESS were more frequently men (72%, *n* = 2537). The median age at the index was 55 years (IQR: 42–65), although patients with at least two ESS revisions were younger (median 48 years, IQR: 35–57) compared with the group without revision surgery (median 56 years, IQR: 44–66, *p*‐value <0.001).

**TABLE 1:** The main baseline characteristics of Finnish adult patients with chronic rhinosinusitis with nasal polyps (CRSwNP) and at least one recorded endoscopic sinus surgery (ESS) during the index period (1st of January 2012 ‐ 31st of December 2018).

Characteristic	ESS done, *N* = 3506	No revisions, *N* = 2794 (79.7%)	One revision, *N* = 559 (15.9%)	At least two revisions, *N* = 153 (4.4%)	^a^ *p*‐value, no revisions versus one revisions	^a^ *p*‐value, no revisions versus at least two revisions	^a^ *p*‐value, one revision versus at least two revisions
Male	2537 (72%)	2051 (73%)	385 (69%)	101 (66%)	0.028	0.045	0.500
Age					<0.001	<0.001	0.120
Mean (SD)	54 (16)	55 (15)	49 (15)	47 (15)			
Median (IQR)	55 (42, 65)	56 (44, 66)	50 (38, 61)	48 (35, 57)			
Follow‐up time (years)					<0.001	<0.001	<0.001
Mean (SD)	4.33 (1.95)	4.20 (1.94)	4.62 (1.91)	5.66 (1.71)			
Median (IQR)	4.30 (2.70, 5.96)	4.14 (2.62, 5.79)	4.67 (3.02, 6.28)	6.07 (4.54, 6.99)			
Follow‐up time (days)					<0.001	<0.001	<0.001
Mean (SD)	1581 (712)	1533 (708)	1689 (697)	2069 (623)			
Median (IQR)	1571 (985, 2177)	1513 (957, 2114)	1707 (1,102, 2294)	2218 (1,659, 2553)			
Asthma at baseline	726 (21%)	514 (18%)	152 (27%)	60 (39%)	<0.001	<0.001	0.004
Asthma severity at baseline					<0.001	<0.001	0.008
No asthma	2780 (79%)	2280 (82%)	407 (73%)	93 (61%)			
Mild to moderate asthma	634 (18%)	448 (16%)	131 (23%)	55 (36%)			
Severe asthma	92 (2.6%)	66 (2.4%)	21 (3.8%)	5 (3.3%)			
INCS at baseline (1 year)	3011 (86%)	2377 (85%)	497 (89%)	137 (90%)	0.018	0.130	0.800
Regular use of INCS at baseline (1 year)	1343 (38%)	1022 (37%)	243 (43%)	78 (51%)	0.002	<0.001	0.100
OCS use at baseline (1 year)	1766 (50%)	1361 (49%)	308 (55%)	97 (63%)	0.006	<0.001	0.066
OCS purchases at baseline (1 year)					<0.001	<0.001	0.004
Mean (SD)	0.81 (1.09)	0.76 (1.04)	0.95 (1.18)	1.29 (1.40)			
Median (IQR)	1.00 (0.00, 1.00)	0.00 (0.00, 1.00)	1.00 (0.00, 1.00)	1.00 (0.00, 2.00)			
OCS daily dose (mg) at baseline (1 year)					0.017	<0.001	0.042
Mean (SD)	2.53 (2.07)	2.45 (2.01)	2.69 (1.98)	3.17 (2.80)			
Median (IQR)	1.64 (1.64, 3.29)	1.64 (1.64, 3.29)	1.64 (1.64, 3.29)	3.29 (1.64, 3.70)			
OCS daily dose (mg) during follow‐up					<0.001	<0.001	<0.001
Mean (SD)	1.36 (1.81)	1.23 (1.83)	1.50 (1.72)	2.01 (1.76)			
Median (IQR)	0.73 (0.36, 1.56)	0.62 (0.32, 1.32)	0.85 (0.44, 1.90)	1.57 (0.76, 2.68)			
(Missing)	1914 (54.6%)	1765 (63.2%)	131 (23.4%)	18 (11.8%)			
Antibiotics use at baseline (1 year)	1979 (56%)	1501 (54%)	361 (65%)	117 (76%)	<0.001	<0.001	0.006
Antibiotics purchases at baseline (1 year)					<0.001	<0.001	<0.001
Mean (SD)	1.15 (1.48)	1.06 (1.42)	1.41 (1.52)	1.98 (1.97)			
Median (IQR)	1.00 (0.00, 2.00)	1.00 (0.00, 2.00)	1.00 (0.00, 2.00)	2.00 (1.00, 3.00)			
Biologics use at baseline (1 year)	0 (0%)	0 (0%)	0 (0%)	0 (0%)			
Biologics use during follow‐up	<5 (<0.1%)	<5 (<0.2%)	<5 (<0.9%)	<5 (<3.3%)	0.030	0.008	0.120
ESS count					<0.001	<0.001	<0.001
1	2794 (80%)	2794 (100%)	0 (0%)	0 (0%)			
2	559 (16%)	0 (0%)	559 (100%)	0 (0%)			
3	100 (2.9%)	0 (0%)	0 (0%)	100 (65%)			
4	34 (1.0%)	0 (0%)	0 (0%)	34 (22%)			
5+	19 (0.5%)	0 (0%)	0 (0%)	19 (12%)			
Time to ESS revision (days)							0.034
Mean (SD)	631 (552)	NA (NA)	663 (581)	513 (408)			
Median (IQR)	425 (213, 898)	NA (NA, NA)	441 (217, 969)	384 (204, 722)			
Extensive surgery (at index)	25 (0.7%)	13 (0.5%)	5 (0.9%)	7 (4.6%)	0.200	<0.001	0.006
Extensive surgery (at index or during the follow‐up)	97 (2.8%)	16 (0.6%)	48 (8.6%)	33 (22%)	<0.001	<0.001	<0.001

*Note*: Patients were further divided into those without revision, one revision and at least two revisions during the follow‐up from the first surgery during the index period until 31st of December 2019.

Abbreviations: INCS, intranasal corticosteroids; IQR, interquartile range; OCS, oral corticosteroids; SD, standard deviation.

^a^ Wilcoxon rank sum test was used for continuous variables, and Chi‐square test for categorical variables.

A total of 86% (*n* = 3011) of the cohort had received INCS within 1 year prior to the baseline. Regular INCS use at baseline was most common in CRSwNP patients with at least two ESS revisions (51%, *n* = 78), compared with patients without revisions (37%, *n* = 1,022, *p*‐value <0.001). Overall, 50% (*n* = 1766) of the CRSwNP patients who had undergone at least one ESS had received OCS at baseline, with a higher frequency (63%, *n* = 97) and median daily dose (3.29 mg, IQR: 1.64–3.70) shown among patients with at least two ESS revisions, compared with patients without revisions (49%, *n* = 1361 and 1.64 mg, IQR: 1.64–3.29, respectively. *p*‐value <0.001). During follow up the median daily OCS dose was reduced across all patients; however, the dose remained high, particularly in patients undergoing at least two ESS revisions (median daily dose 1.57 mg, IQR: 0.76, 2.68). Similarly, antibiotic use at baseline was more common in CRSwNP patients with at least two ESS revisions (76%, *n* = 117) compared with patients without revisions (54%, *n* = 1,501, *p*‐value < 0.001). None of the CRSwNP patients received biological therapy at baseline, yet exposure to biologics during the follow‐up was more common in patients with at least two revisions (<3.3%, *n* ≤ 5) compared with patients without revisions (<0.2%, *n* ≤ 5, *p*‐value 0.008). Extensive surgery at index (4.6%, *n* = 7) and extensive surgery during the follow‐up (22.0%, *n* = 33) were more frequent among CRSwNP patients with at least two ESS revisions compared with patients without revision surgery (0.5% and 0.6%, respectively. *p*‐value <0.001).

### 3.1 Comorbidities

Overall, 80% of the patients who had undergone ESS once (i.e., without revision surgery) had a CCI score of 0, followed by patients with one revision (75%) and patients with at least two revisions (73%) (Table 2). The most prominent differences were observed for the CCI score of 1, with 23% and 21% in the groups of at least two revisions and one revision, respectively, whilst 14% of patients without a revision surgery had a CCI score of 1.

**TABLE 2:** Charlson comorbidity index (CCI) scores and the included comorbidities at baseline, stratified by the endoscopic sinus surgery (ESS) status.

Characteristic	ESS done, *N* = 3506	No revisions, *N* = 2794 (79.7%)	One revision, *N* = 559 (15.9%)	At least two revisions, *N* = 153 (4.4%)
CCI				
0	2753 (79%)	2222 (80%)	419 (75%)	112 (73%)
1	534 (15%)	379 (14%)	120 (21%)	35 (23%)
2	142 (4.1%)	123 (4.4%)	14 (2.5%)	5 (3.3%)
3	57 (1.6%)	51 (1.8%)	5 (0.9%)	<5 (<3.3%)
4	10 (0.3%)	9 (0.3%)	<5 (<0.9%)	0 (0%)
5	<5 (<0.1%)	<5 (<0.2%)	0 (0%)	0 (0%)
5+	7 (0.2%)	7 (0.3%)	0 (0%)	0 (0%)
Chronic pulmonary disease	578 (16.5%)	418 (15%)	124 (22.2%)	36 (23.5%)
Cancer (any malignancy)	138 (3.9%)	123 (4.4%)	10 (1.8%)	5 (3.3%)
Cerebrovascular disease	88 (2.5%)	80 (2.9%)	8 (1.4%)	0 (0.0%)
Congestive heart failure	39 (1.1%)	31 (1.1%)	8 (1.4%)	0 (0.0%)
Peripheral vascular disease	33 (0.9%)	30 (1.1%)	*n* < 5 (<0.9%)	*n* < 5 (<3.3%)
Dementia	29 (0.8%)	28 (1%)	*n* < 5 (<0.9%)	0 (0.0%)
Rheumatoid disease	24 (0.7%)	20 (0.7%)	*n* < 5 (<0.9%)	0 (0.0%)
Acute myocardial infarction	21 (0.6%)	15 (0.5%)	6 (1.1%)	0 (0.0%)
Renal disease	16 (0.5%)	15 (0.5%)	*n* < 5 (<0.9%)	0 (0.0%)
Hemiplegia or paraplegia	8 (0.2%)	7 (0.3%)	0 (0.0%)	*n* < 5 (<3.3%)
Peptic ulcer disease	8 (0.2%)	6 (0.2%)	*n* < 5 (<0.9%)	0 (0.0%)
Metastatic solid tumor	5 (0.1%)	5 (0.2%)	0 (0.0%)	0 (0.0%)
Mild liver disease	5 (0.1%)	5 (0.2%)	0 (0.0%)	0 (0.0%)

Of the CCI score included comorbidities, the top three most frequently occurring diseases were chronic pulmonary disease (16.5%), cancer (3.9%), and cerebrovascular disease (2.5%). The greatest difference across the groups was noted among individuals with chronic pulmonary disease, which was more frequent among patients with at least two revisions and one revision ESS (23.5% and 22.5%, respectively) compared with patients without revisions (15%).

Altogether, 18% (*n* = 634) of the CRSwNP patients who had undergone ESS had been diagnosed with mild to moderate asthma at baseline. Again, this was more common among patients with at least two surgery revisions (36%, *n* = 55), compared with patients without revision surgery (16%, *n* = 448, *p*‐value <0.001). Severe asthma (at baseline) was diagnosed in 2.6% (*n* = 92) of the patients (Table S4).

### 3.2 Revision surgery within 1 year

Of the CRSwNP patients who underwent revision ESS, 10.2% (*n* = 322) had revision surgery within the first year from the index. Patients who had undergone extensive surgery at index had the highest odds of revision ESS within 1 year (adjusted OR = 3.33, 95% CI 1.19–8.09) compared with patients who had undergone limited surgery based on point estimates (Table 3). Compared to CRSwNP patients without exposure to antibiotics at baseline, patients exposed to antibiotics had higher odds of revision ESS within 1 year (aOR = 1.58, 95% CI 1.23–2.03). In contrast, patients with OCS use at baseline had lower odds of revision ESS within 1 year (aOR = 0.69, 95% CI 0.53–0.89) compared with patients without OCS use. A marginal association was noted for baseline asthma (aOR = 1.40, 95% CI 1.00–1.92) and increasing median age (every 10 years) (aOR = 0.93, 95% CI 0.86–1.00), although with different directions of association. No significant association was found for INCS regular use at baseline or CCI score. When assessing relative variable importance, exposure to antibiotics at baseline was the most important predictor of revision ESS within 1 year with a mean Shapley value (absolute log odds scale) of 0.222. This indicates that antibiotic exposure had the strongest effect on increasing the likelihood of revision of ESS. Other key predictors were exposure to OCS, age and baseline asthma with mean Shapley values of 0.188, 0.131, and 0.108, respectively. An analysis of VIFs and pairwise correlations among the independent variables indicated that multicollinearity was not a significant issue in our dataset, as all VIFs were below 5 and all correlation coefficients (|r|) were less than 0.5.

**TABLE 3:** The odds of revision endoscopic sinus surgery (ESS) within 1 year from the index, providing odds ratios (OR) with corresponding 95% confidence intervals (CIs) based on logistic regression.

Variable		No revision ESS, *N* = 3151	Revision ESS, *N* = 322 (10.2%)	OR (univariable)	OR (multivariable)	Shapley
Age (10‐years)	Mean (SD)	5.4 (1.6)	5.2 (1.5)	0.92 (0.86–0.99, *p* = 0.031)	0.93 (0.86–1.00, *p* = 0.053)	0.131
Sex	Female	866 (27.5)	96 (29.8)	‐	‐	0.014
	Male	2285 (72.5)	226 (70.2)	0.89 (0.70–1.15, *p* = 0.374)	0.97 (0.75–1.26, *p* = 0.793)	
Antibiotics 1‐year baseline	No antibiotics	1393 (44.2)	111 (34.5)	‐	‐	0.222
	Antibiotics	1758 (55.8)	211 (65.5)	1.51 (1.19–1.92, *p* = 0.001)	1.58 (1.23–2.03, *p* < 0.001)	
OCS 1‐year baseline	No OCS	1542 (48.9)	178 (55.3)	‐	‐	0.188
	OCS	1609 (51.1)	144 (44.7)	0.78 (0.62–0.98, *p* = 0.030)	0.69 (0.53–0.89, *p* = 0.004)	
INCS regular use at baseline	No INCS regular use	1926 (61.1)	211 (65.5)	‐	‐	0.082
	INCS regular use	1225 (38.9)	111 (34.5)	0.83 (0.65–1.05, *p* = 0.122)	0.84 (0.65–1.09, *p* = 0.198)	
Type of surgery at index	Limited surgery	3132 (99.4)	316 (98.1)	‐	‐	0.015
	Extensive surgery	19 (0.6)	6 (1.9)	3.13 (1.13–7.46, *p* = 0.016)	3.33 (1.19–8.09, *p* = 0.012)	
CCI score at baseline	CCI 0	2482 (78.8)	258 (80.1)	‐	‐	0.066
	CCI 1+	669 (21.2)	64 (19.9)	0.92 (0.69–1.22, *p* = 0.570)	0.82 (0.58–1.15, *p* = 0.265)	
Asthma at baseline	No asthma	2508 (79.6)	246 (76.4)	‐	‐	0.108
	Asthma	643 (20.4)	76 (23.6)	1.21 (0.91–1.57, *p* = 0.178)	1.40 (1.00–1.92, *p* = 0.044)	

Abbreviations: CCI, Charlson comorbidity index; INCS, intranasal corticosteroids; OCS, oral corticosteroids; SD, standard deviation.

### 3.3 Revision surgery within 3 years

Of the CRSwNP patients who underwent revision ESS, 20.9% (*n* = 421) had revision surgery within 3 years from the index. Patients who had undergone extensive surgery at the index had the highest odds of revision ESS within 3 years (aOR = 14.13, 95% CI 3.41–95.64) compared to patients who had undergone limited surgery, based on a smaller group (Table 4). Similarly, patients who had been exposed to antibiotics at baseline (aOR = 1.61, 95% CI 1.27–2.04) and patients with asthma (aOR = 1.58, 95% CI 1.17–2.12) had higher odds of revision ESS at 3 years, compared to patients without antibiotic exposure and asthma, respectively. In contrast, increasing mean age (10 years) was associated with lower odds of revision ESS (aOR = 0.82, 95% CI 0.76–0.88). No statistically significant association was found for OCS use, INCS regular use at baseline, or CCI score. The most important predictors of revision ESS within 3 years were age and exposure to antibiotics at baseline with mean Shapley values of 0.351 and 0.231, respectively. Excluding extensive surgery from the models had close to no impact on the predictors of revision surgery at one or 3 years (data not shown).

**TABLE 4:** The odds of revision endoscopic sinus surgery (ESS) within 3 years from the index, providing odds ratios (OR) with corresponding 95% confidence intervals (CIs) based on logistic regression.

Variable		No revision ESS, *N* = 2013	Revision ESS, *N* = 421 (20.9%)	OR (univariable)	OR (multivariable)	Shapley
Age (10‐years)	Mean (SD)	5.4 (1.5)	4.9 (1.5)	0.81 (0.76–0.87, *p* < 0.001)	0.82 (0.76–0.88, *p* < 0.001)	0.351
Sex	Female	555 (27.6)	131 (31.1)	‐	‐	0.005
	Male	1458 (72.4)	290 (68.9)	0.84 (0.67–1.06, *p* = 0.142)	0.99 (0.78–1.26, *p* = 0.923)	
Antibiotics 1‐year baseline	No antibiotics	895 (44.5)	128 (30.4)	‐	‐	0.231
	Antibiotics	1118 (55.5)	293 (69.6)	1.83 (1.47–2.30, *p* < 0.001)	1.61 (1.27–2.04, *p* < 0.001)	
OCS 1‐year baseline	No OCS	1039 (51.6)	183 (43.5)	‐	‐	0.006
	OCS	974 (48.4)	238 (56.5)	1.39 (1.12–1.72, *p* = 0.002)	1.01 (0.80–1.28, *p* = 0.919)	
INCS regular use at baseline	No INCS regular use	1287 (63.9)	237 (56.3)	‐	‐	0.084
	INCS regular use	726 (36.1)	184 (43.7)	1.38 (1.11–1.70, *p* = 0.003)	1.20 (0.95–1.51, *p* = 0.135)	
Type of surgery at index	Limited surgery	2011 (99.9)	413 (98.1)	‐	‐	0.017
	Extensive surgery	2 (0.1)	8 (1.9)	19.48 (4.86–129.35, *p* < 0.001)	14.13 (3.41–95.64, *p* = 0.001)	
CCI score at baseline	CCI 0	1613 (80.1)	319 (75.8)	‐	‐	0.002
	CCI 1+	400 (19.9)	102 (24.2)	1.29 (1.00–1.65, *p* = 0.045)	1.01 (0.74–1.37, *p* = 0.969)	
Asthma at baseline	No asthma	1624 (80.7)	296 (70.3)	‐	‐	0.150
	Asthma	389 (19.3)	125 (29.7)	1.76 (1.39–2.23, *p* < 0.001)	1.58 (1.17–2.12, *p* = 0.002)	

Abbreviations: CCI, Charlson comorbidity index; INCS, intranasal corticosteroids; OCS, oral corticosteroids; SD, standard deviation.

### 3.4 Association of antibiotics and asthma with the probability of revision surgery

Based on the logistic regression prediction model, an average patient in the cohort, a 55‐year‐old male without asthma and a history of antibiotic use, had an 11% probability (95% CI 8%–13%) of revision ESS within three years from the index (Table 5). If the patient had either asthma or a history of antibiotic use, the predicted probability of revision of ESS increased to 16% (95% CI 11%–21% and 13%–19%, respectively). The highest predicted probability of a revision ESS was observed for an average person with both asthma and a history of antibiotic use (23%, 95% CI 17%–30%).

**TABLE 5:** Impact of antibiotics and asthma on the probability of a revision endoscopic sinus surgery (ESS) within 3 years from the index in an average male patient without the use of oral or intranasal corticosteroids, limited surgery at baseline, average 16.5 annual contacts and a Charlson comorbidity index score of zero.

Average patient Male, age (years)	Antibiotics	Asthma	^a^Revision ESS probability
55	No antibiotics	No asthma	0.11 (0.08–0.13)
55	No antibiotics	Asthma	0.16 (0.11–0.21)
55	Antibiotics	No asthma	0.16 (0.13–0.19)
55	Antibiotics	Asthma	0.23 (0.17–0.30)

^a^ Probability was calculated by bootstrapping, 10 10,000 samples.

## 4 DISCUSSION

This nationwide population‐based study utilizing Finnish real‐world data explored the association of several patient characteristics of CRSwNP with the likelihood of revision of ESS. We found that asthma and antibiotic use at baseline were associated with higher odds of revision of ESS within 1–3 years post‐surgery. The highest odds for revision of ESS were observed among patients who had undergone extensive surgery at index. Potentially this reflects differences in the underlying severity of the disease; however, due to limited patient numbers, the results should be interpreted with caution. In contrast, an inverse association was shown for increasing mean age at the index. Furthermore, OCS use at baseline was associated with lower odds of revision ESS within 1 year but not within 3 years from the index. This could indicate that OCS treatment has an adequate short‐term effect in controlling CRS and associated symptoms, but the benefit does not persist and therefore does not prevent future revisions.

The observed ESS revision rate (15.9%) is mostly consistent with previous studies. In a recent meta‐analysis, including 34,200 individuals, a mean revision rate of 16.2% over a weighted mean follow‐up time of 89.6 months was reported,^18^ whereas in other retrospective cohort studies, revision rates of approximately 9% and 25% were observed.^29^, ^30^ The slight discrepancy in the revision rates could be explained by different definitions, study design, settings, and length of follow‐up time, hampering any direct comparison. Notably, we found that patients with more revision surgeries had a longer median follow‐up time compared to those with one revision or no revision surgery, with the longest median follow‐up time observed in patients with ≥2 revisions (6.07 years), reflecting the progression of the disease.

Similar to our findings, where 21% of patients had an asthma diagnosis at baseline, the meta‐analysis suggested that asthma (22.6%) was associated with increased revision rates.^18^ A recent registry‐based study conducted in the Finnish Hospital District of Helsinki and Uusimaa reported asthma comorbidity in a much larger proportion of CRSwNP patients (48.6%)^9^ This discrepancy could be due to the more stringent criteria for asthma implemented in our study and/or the shorter duration of follow‐up, as the cumulative proportion of asthma is likely to increase over time.

In our study, adherence to standard‐of‐care treatment was low, with less than 40% of patients taking regular INCS in the 1 year prior to baseline. OCS use, on the other hand, was frequent (50% at baseline) with a higher cumulative dose in patients undergoing multiple ESS revisions. Although a reduction in OCS use was observed during follow‐up, the cumulative OCS dose remained high, particularly in patients undergoing revision surgeries. This is in contrast with clinical recommendations stating that OCS short courses should only be considered as a last resort of treatment when combinations of other medications are ineffective.^31^

Although the cumulative risks of OCS are well known, until recently, two OCS courses per year were considered acceptable for CRSwNP patients treated in primary and specialty care.^32^, ^33^ However, when the disease is chronic, a cumulative dose of over 1g of steroids can be achieved already in 2 years, exceeding the OCS exposure associated with adverse outcomes^32^, ^34^, ^35^ Several biological treatments have become available targeting the type 2 inflammatory response in patients with CRSwNPs. These have been shown to improve symptom control and reduce corticosteroid burden in patients where systemic corticosteroids and/or surgery have not achieved adequate disease control, thus offering a new therapeutic option for such severe patients.^36^, ^37^, ^38^, ^39^, ^40^

The main strength of this study is the nationwide and population‐based approach, capturing virtually the entire Finnish population of 5.5 million individuals since 2012. This counteracts the risk of selection bias, ensures statistical power, and enhances the generalizability of the results. Data on reimbursed outpatient care drug purchases is complete, and underestimation of exposure to antibiotics or OCS is unlikely, given these are only available on prescription in Finland. INCSs, on the other hand, are also available over‐the‐counter and INCS exposure may thus be underreported. Over‐the‐counter use is nevertheless expected to be low as it is more affordable to obtain the drugs on prescription.

A limitation of the study was the inclusion of patients with incident nasal polyps only. Considering the maximum follow‐up time of 8 years, we may have captured only a sub‐population of patients with a faster rate of disease development. Furthermore, although comorbidities were identified by ICD‐10 codes in specialty and primary care, data on the occupational health care codes were lacking. Also, the practice of ICD‐10 coding among clinicians might vary, and thus, some codes could have been misclassified.

In addition, we noticed differences in recording practices of surgical procedures across the various hospitals. In some areas, visits with ESS codes were more frequent and occurred at short intervals, likely reflecting visits due to complications, control visits, or coding errors. To avoid a potential overestimation of the frequency of actual ESS revisions, a conservative approach was chosen, and a cut‐off limit of 90 days between ESS visits was applied.

Another potential concern is confounding by indication. This may be particularly relevant for the found association between antibiotic use and revision ESS, as antibiotics included in this study are specifically used for treating sinusitis, and recurrent antibiotic courses are an indication for surgery. Further logistic regression assumes a linear relationship between the predictors and the log‐ratios of the outcome and might underperform if the true relationship between revision ESS and the predictors is more complex. On the other hand, overfitting due to multiple variables can affect the generalizability of the models, which should be kept in mind when interpreting the results.

Lastly, we lacked data on potential biomarkers, for example, blood eosinophils, to detect distinct clinicopathological variants of the disease. Considering the multidimensional heterogeneity of the disease, it is likely that some endotypes are more difficult to treat or could potentially have poorer response to treatment.^1^, ^2^ Although ESS adds value to the treatment of CRS among most of the patients,^16^, ^41^ several surgeries might reflect differences in the severity of the underlying endotype and/or uncontrolled disease, also highlighted by the increase of comorbid asthma.

In conclusion, this nationwide study demonstrates that a small proportion of CRSwNP patients require multiple ESS revision surgeries to manage their disease. OCS and antibiotic use among these patients is frequent, and the cumulative OCS dose remains high post‐surgery. This highlights the need for effective therapies, such as biologics, to achieve disease control and reduce the OCS and antibiotic burden in these patients.

## AUTHOR CONTRIBUTIONS

Sanna Toppila‐Salmi, Lauri Lehtimäki, Annina Lyly, Juhani Aakko, Johanna Simin and Helga Haugom Olsen were involved in the concept and design. Juhani Aakko conducted the statistical analyses. All authors, Sanna Toppila‐Salmi, Annina Lyly, Johanna Simin, Juhani Aakko, Helga Haugom Olsen and Lauri Lehtimäki, contributed to the interpretation of the findings, were actively involved in the manuscript writing and critically revised it. All authors, Sanna Toppila‐Salmi, Annina Lyly, Johanna Simin, Juhani Aakko, Helga Haugom Olsen and Lauri Lehtimäki, gave their final approval for manuscript publication and agreed to be accountable for all aspects of their work. The study was funded by AstraZeneca Nordic.

## CONFLICT OF INTEREST STATEMENT

HHO is an employee of AstraZeneca, the study Sponsor, and may have stock ownership and/or stock options or interests in the company. AL was an employee of AstraZeneca during the completion of the study and is currently employed at the Helsinki University Hospital. STS reports consultancies for ALK‐Abelló, AstraZeneca, Clario, GSK, Novartis, Orion Pharma, Sanofi, and Roche Products outside the submitted work as well as grant of GSK and Sanofi outside the submitted work.

## Supporting information

Supporting Information S1

## Data Availability

The dataset from this study is held at Findata's secure operating environment, Kapseli, yet data sharing agreements prohibit making the dataset publicly available. The data and the underlying analysis plan are, however, available from the authors upon a reasonable request to the corresponding author (Sanna Toppila‐Salmi) and after obtaining the relevant permissions from Finnish Authorities.
