# Supplementary material for: Predictors of revision endoscopic sinus surgery in Finnish patients with chronic rhinosinusitis with nasal polyps
Source: Clin Transl Allergy. 2025 Jan 29;15(2):e70032. doi: 10.1002/clt2.70032 (PMC11779534; doi:10.1002/clt2.70032)
Supplement: Supplementary file 1 — Supporting Information S1 [file CLT2-15-e70032-s001.docx]

**Supplementary Materials and Tables**

- **S1 Table**: The ICD-10, ICPC-2 and NOMESCO Classification of Surgical Procedures (NCSP) codes considered in this study.
- **S2 Table:** Baseline exposure definitions and applied classification codes.
- **S3 Table:** Outcome procedure codes.
- **S4 Table:** The most frequently occurring medical baseline diagnoses (based on ICD-10 codes), stratified by ESS status.

**S1**: The ICD-10, ICPC-2 and NOMESCO Classification of Surgical Procedures (NCSP) codes considered in this study.

| **Code system** | **Description** |
| --- | --- |
| **ICD-10** |  |
| J32.0 | Chronic maxillary sinusitis* |
| J32.1 | Chronic frontal sinusitis* |
| J32.2 | Chronic ethmoidal sinusitis* |
| J32.3 | Chronic sphenoidal sinusitis* |
| J32.4 | Chronic pansinusitis* |
| J32.8 | Other chronic sinusitis* |
| J32.9 | Chronic sinusitis, unspecified* |
| J33.0 | Polyp of nasal cavity |
| J33.1 | Polypoid sinus degeneration |
| J33.8 | Other nasal polyp |
| J33.9 | Undefined nasal polyp |
| **NCSP** |  |
| DHB20 | Polypectomy of internal nose |
| DMB00 | Endonasal trephine of maxillary antrum |
| DMB10 | Radical maxillary antrotomy |
| DMB20 | Functional endoscopic opening of maxillary antrum |
| DMW99 | Other operation on maxillary antrum |
| DNB20 | Endoscopic ethmoidectomy |
| DPA20 | Trephine of frontal sinus |
| DPA25 | Trephine of frontal sinus through nose |
| DPA30 | Sphenotomy |
| ZXC95 | Intracranial endoscopic technique |

*Combined with NCSP code for polypectomy: DHB20 (within two years from the diagnosis).

**S2:** Baseline exposure definitions.

| **Definition** | **Description** |
| --- | --- |
| Diagnosis of asthma (based on ICD-10 codes and ATC codes) | At least two recorded visits with an asthma diagnosis (ICD-10 code J45) or at least two asthma controller medication purchases (ICS) in single inhalers (ATC-codes R03BA01, R03BA02, R03BA05, R03BA07, R03BA08, R03BA09) or in combination with long-acting beta-2-agonists (R03AK06, R03AK07, R03AK08, R03AK09, R03AK10, R03AK11). |
| Severe asthma | Severe asthma was assessed based on drug purchases (data from SII). Severe asthma was defined as asthma with a daily use of fluticasone propionate >= 800 µg or equivalent (80% adherence to 1000 µg or equivalent) complemented with at least one other controller (LTRA, LABA, LAMA, or biologic medication). The daily average inhaled corticosteroid (ICS) use was calculated based on a sliding window of three consecutive purchases (the total micrograms of ICs at three consecutive purchases divided by days from the first purchase to the fourth consecutive purchase). |
| Antibiotics for sinusitis (ATC codes) | - J01CE02: penicillin - J01CA04: amoxicillin - J01CR02: amoxicillin + beta-lactamase inhibitor - J01AA02: doxycycline - J01E: sulfonamides and trimethoprim - J01FA10: azithromycin - J01DB01: cefalexin |
| Other relevant medications considered in the study (ATC codes) | - R01* intranasal steroids - H02* systemic corticosteroids - R03* asthma medications (inhaled and biologics)   *Including all medications within this group on the most specific ATC code level in the hierarchy. |
| Days covered by intranasal steroid purchases | The days covered by intranasal steroid purchases were calculated by dividing the total dose of intra-nasal steroid purchases during the one-year baseline by half of the respective defined daily dose (DDD). |
| Regular use of intra-nasal steroids | The one-year baseline intranasal steroid purchases cover > 270 days. |
| Extensive surgery at index | DNB20 together with ZXC95 on the same day or DNB20, DPA30, and DPA25 on the same day.* |

* DNB20 = Endoscopic Ethmoidectomy: Opening of the ethmoidal cells (small air-filled spaces between the nose and eyes) to improve drainage.

DPA30 = Sphenotomy: Opening of the sphenoid sinus, a deep sinus located behind the eyes.

DPA25 = Trephine of the Frontal Sinus: Creating an opening in the frontal sinus (located in the forehead) through the nasal passage.

ZXC95 = Navigator endoscopic technique.

Surgeries not meeting these criteria were classified as limited ESS.

**S3:** Outcome procedure codes.

| **Definition** | **Description** |
| --- | --- |
| Endoscopic sinus surgery for CRSwNP | Procedure codes:  DHB20 Polypectomy of internal nose; DMB00 Endonasal trephine of maxillary antrum; DMB10 Radical maxillary antrotomy; DMB20 Functional endoscopic opening of maxillary antrum; DMW99 Other operation on maxillary antrum; DNB20 Endoscopic ethmoidectomy; DPA20 Trephine of frontal sinus; DPA25 Trephine of frontal sinus through nose; DPA30 Sphenotomy  SPAT2: SPAT1023 Polypectomy |
| Revision endoscopic sinus surgery | Any of the procedure codes for ESS > 90 days after the previously recorded end of an ESS episode. The start of an ESS episode is defined as the first visit where an ESS procedure code was recorded. Subsequent visits that occur within 90 days from the previous visit will be merged into the same episode. The end of an ESS episode is defined as the last visit where an ESS procedure was recorded, followed by a period of at least 90 days before the next visit with an ESS procedure. |

**S4:** The 25 most frequent co-diagnoses on top of J32/J33 at baseline (based on ICD-10 codes), stratified by endoscopic sinus surgery (ESS) status.

| **ICD-10** | **Description** | **ESS done,**  **n = 3,506** | **No revisions,**  **n = 2,794** | **One revision,**  **n = 559** | **At least two revisions,**  **n = 153** |
| --- | --- | --- | --- | --- | --- |
| K02 | Dental caries | 632 (18%) | 492 (17.6%) | 116 (20.8%) | 24 (15.7%) |
| J01 | Acute sinusitis | 629 (17.9%) | 468 (16.8%) | 125 (22.4%) | 36 (23.5%) |
| J45 | Asthma | 502 (14.3%) | 355 (12.7%) | 114 (20.4%) | 33 (21.6%) |
| I10 | Essential (primary) hypertension | 466 (13.3%) | 393 (14.1%) | 60 (10.7%) | 13 (8.5%) |
| J06 | Acute upper respiratory infections of multiple and unspecified sites | 336 (9.6%) | 249 (8.9%) | 68 (12.2%) | 19 (12.4%) |
| M54 | Dorsalgia | 292 (8.3%) | 232 (8.3%) | 47 (8.4%) | 13 (8.5%) |
| K04 | Diseases of pulp and periapical tissues | 285 (8.1%) | 215 (7.7%) | 61 (10.9%) | 9 (5.9%) |
| J34 | Other disorders of nose and nasal sinuses | 280 (8%) | 232 (8.3%) | 38 (6.8%) | 10 (6.5%) |
| K03 | Other diseases of hard tissues of teeth | 241 (6.9%) | 181 (6.5%) | 49 (8.8%) | 11 (7.2%) |
| R06 | Abnormalities of breathing | 243 (6.9%) | 180 (6.4%) | 48 (8.6%) | 15 (9.8%) |
| R10 | Abdominal and pelvic pain | 214 (6.1%) | 158 (5.7%) | 48 (8.6%) | 8 (5.2%) |
| G47 | Sleep disorders | 209 (6%) | 172 (6.2%) | 35 (6.3%) | n < 5 (< 3.3%) |
| J30 | Vasomotor and allergic rhinitis | 208 (5.9%) | 156 (5.6%) | 41 (7.3%) | 11 (7.2%) |
| J20 | Acute bronchitis | 202 (5.8%) | 151 (5.4%) | 40 (7.2%) | 11 (7.2%) |
| J31 | Chronic rhinitis, nasopharyngitis and pharyngitis | 198 (5.6%) | 153 (5.5%) | 34 (6.1%) | 11 (7.2%) |
| E11 | Non-insulin-dependent diabetes mellitus | 193 (5.5%) | 164 (5.9%) | 25 (4.5%) | n < 5 (< 3.3%) |
| E78 | Disorders of lipoprotein metabolism and other lipidaemias | 192 (5.5%) | 164 (5.9%) | 23 (4.1%) | 5 (3.3%) |
| K05 | Gingivitis and periodontal diseases | 181 (5.2%) | 146 (5.2%) | 29 (5.2%) | 6 (3.9%) |
| M79 | Other soft tissue disorders, not elsewhere classified | 172 (4.9%) | 140 (5%) | 25 (4.5%) | 7 (4.6%) |
| M75 | Shoulder lesions | 161 (4.6%) | 136 (4.9%) | 23 (4.1%) | n < 5 (< 3.3%) |
| M17 | Gonarthrosis [arthrosis of knee] | 139 (4%) | 111 (4%) | 21 (3.8%) | 7 (4.6%) |
| N40 | Hyperplasia of prostate | 135 (3.9%) | 118 (4.2%) | 12 (2.1%) | 5 (3.3%) |
| R07 | Pain in throat and chest | 133 (3.8%) | 106 (3.8%) | 25 (4.5%) | n < 5 (< 3.3%) |
| S02 | Fracture of skull and facial bones | 125 (3.6%) | 97 (3.5%) | 25 (4.5%) | n < 5 (< 3.3%) |
